# Process Evaluation of a Workers’ Health Surveillance Program for Meat Processing Workers

**DOI:** 10.1007/s10926-016-9657-y

**Published:** 2016-07-30

**Authors:** Berry J. van Holland, Sandra Brouwer, Michiel R. de Boer, Michiel F. Reneman, Remko Soer

**Affiliations:** 1 Department of Rehabilitation Medicine, Center for Rehabilitation, University Medical Center Groningen, University of Groningen, Hanzeplein 1, 9713 GZ Groningen, The Netherlands; 2 Department of Health Sciences, Community and Occupational Medicine, University Medical Center Groningen, University of Groningen, Groningen, The Netherlands; 3 Department of Health Sciences, Faculty of Earth and Life Sciences, Institute for Health Sciences, VU University, Amsterdam, The Netherlands; 4 Expertise Center of Health, Social Care and Technology, Saxion University of Applied Sciences, Enschede, The Netherlands; 5 Groningen Spine Center, University Medical Center Groningen, University of Groningen, Groningen, The Netherlands

**Keywords:** Implementation research, Meatpacking industry, Sustainable employability, Quantitative, Qualitative

## Abstract

*Objective* To evaluate the implementation process of a workers’ health surveillance (WHS) program in a Dutch meat processing company. *Methods* Workers from five plants were eligible to participate in the WHS program. The program consisted of four evaluative components and an intervention component. Qualitative and quantitative methods were used to evaluate seven process aspects. Data were gathered by interviews with stakeholders, participant questionnaires, and from registries of the company and occupational health service. *Results* Two recruitment strategies were used: open invitation or automatic participation. Of the 986 eligible workers, 305 participated in the program. Average reach was 53 %. Two out of five program components could not be assessed on dose delivered, dose received and fidelity. If components were assessable, 85–100 % of the components was delivered, 66–100 % of the components was received by participants, and fidelity was 100 %. Participants were satisfied with the WHS program (mean score 7.6). Contextual factors that facilitated implementation were among others societal developments and management support. Factors that formed barriers were program novelty and delayed follow-up. *Conclusion* The WHS program was well received by participants. Not all participants were offered the same number of program components, and not all components were performed according to protocol. Deviation from protocol is an indication of program failure and may affect program effectiveness.

## Introduction

Several workplace health promotion (WHP) programs have demonstrated effectiveness [1, 2]. Recent reviews showed moderate to strong evidence for positive effects of disability management and return-to-work programs, ergonomic adjustments and training, and participatory ergonomics programs [1]. Furthermore, WHP programs showed a stronger effect in younger populations (<40 years), in interventions with frequent contacts (at least weekly), and in studies with lower methodological quality [2].

Due to an aging workforce and rising retirement age, effective WHP programs for older workers should be developed [3, 4]. Companies are facing workers who indicate that they are unable to continue work until retirement age. Some issues are that older workers are more susceptible to develop chronic health conditions and have longer sick leaves [5, 6]. To help workers reach retirement age in good health, one of the largest meat processing companies in the Netherlands has implemented a novel workers’ health surveillance (WHS) program aiming to promote sustainable employability. WHS is a similar type of intervention as WHP. The introduction of comprehensive health screening and individualized interventions in a WHS program creates the opportunity to address risk factors for reduced health and employability in an early stage [7]. This may allow a more proactive approach in managing and handling possible risk factors and thereby protection of health and employability. However, effectiveness of this WHS program has not yet been investigated. Knowledge about the effectiveness of such programs is valuable, but knowledge about how and why they are (in)effective may help to adapt and revise programs in order to make them more suitable and effective to promote workers’ health [1].

For a WHS program to be effective, two aspects are important: the program must be based on sound rationale, and the program must be implemented and executed according to protocol [8]. Inadequacy of one or both reduces chances that a program will be effective. If a program fails due to a faulty rationale this is called theory failure. If a program fails due to suboptimal implementation this is referred to as program failure [8]. To investigate whether theory and program implementation are adequate a process evaluation can be performed. A process evaluation is a systematic approach that assesses whether a program was implemented according to protocol, how it was implemented, and whether there were circumstances that could have influenced program outcomes. Several previous studies have identified factors that influenced program effectiveness. Factors that were frequently mentioned were contextual barriers and facilitators [9, 10], program reach [9, 10], protocol adherence [9, 11], worker engagement [9], and worker compliance [11, 12].

The objective of this study was to evaluate the implementation process of a WHS program at a large Dutch meat processing company. To structure the evaluation we used the process evaluation framework recommended by Linnan and Steckler [13]. Furthermore, compliance to advices from the WHS program was evaluated.

## Methods

### Study Design

This process evaluation is part of the FLESH study (Functional Labor Evaluation of Sustained Health and employment), a stepped wedge trial evaluating a comprehensive WHS program in the largest Dutch meat processing company [7]. The WHS program was named the promotion of sustained employability (POSE) program. As recommended by the framework described by Linnan and Steckler [13], seven process aspects were evaluated: recruitment, reach, dose delivered, dose received, fidelity, satisfaction, and context. The process aspects were collected by different methods at different levels: company plant (human resource (HR) management, employees), company headquarters, and occupational health service (OHS). Because the study evaluated ‘care as usual’ the Medical Ethics Board of the University Medical Center Groningen decided that formal approval of the study was not necessary. The FLESH study is registered at the Dutch Trial Register (www.trialregister.nl): NTR3445.

### Study Population

Between February 2012 and March 2014 a total of 986 employees from five plants were eligible to participate in the POSE program. The POSE program was implemented at different time points at the involved plants, because of the stepped wedge design we applied, so invitation occurred separately at each plant.

### Intervention

The POSE program has been elaborately described elsewhere [7]. In short, the program consists of four evaluative components (questionnaire, biometric measurements, functional capacity evaluation (FCE), and a counseling session) and an intervention component. The results of the evaluative components indicated whether subsequent intervention was needed. The first evaluative component was an online questionnaire focusing on work ability, health, and lifestyle. The questionnaire could be filled out at home or at work. The second component addressed several biometric features, such as body length, weight, blood pressure, and cholesterol. Those were measured at the workplace by a nurse. The third component, the FCE, was administered by an occupational physiotherapist and addressed material handling, postural tolerance, coordination and repetition, hand and finger strength, and energetic capacity [14, 15]. The fourth component was a counseling session in which the physiotherapist discussed the results of the first three components with the participant. For all POSE program measurements participants were categorized according to a traffic light model. For each outcome, red indicated high risk, orange was medium risk, and green was low or no risk for reduced employability. Based on the traffic light model, participants received advice on follow-up, i.e. whether subsequent interventions were needed, for instance a visit to the family physician, occupational physician, physiotherapist, dietician, or a workplace evaluation.

### Data Collection of Process Outcomes

Quantitative data were collected at baseline and at 3 and 9 months follow-up. At baseline employees enrolled in the POSE program. Various work-related, health-related, and personal characteristics were assessed by questionnaire and measurements. After 3 months, participants were called by the OHS and asked whether they had followed-up on the advice from the POSE program. At 9 months, participants received a questionnaire which evaluated satisfaction with the program, parts of the process, contextual aspects, and program advice (Appendix “Follow-up questionnaire”). This questionnaire was constructed by using elements from the Client Satisfaction Questionnaire (CSQ-8) [16] and the Europep questionnaire [17], supplemented with newly developed questions by the authors.

Qualitative data were collected during semi-structured interviews, which were conducted between November 2014 and January 2015 (Appendix “Interview with HR managers and OHS employees” and “Interview with POSE program participants”). Interviewees were informed about the purpose of the interview, but did not receive further details up front. POSE program participants were selected from the included plants, based on their risk profile. Per plant we aimed to include one worker with low risk, one with medium risk, and one with high risk for sickness absence. Other interviewees were involved HR managers and OHS employees. Interviews were used to provide an in-depth view to the implementation process. Table 1 provides an overview of process outcomes, which are further explained below, and data collection methods.

**Table 1:** Process outcomes and data collection methods

Process outcome	Method	Target group
Recruitment	Interviews	4 HR managers, 10 POSE program participants
Reach	Registry	OHS, Company
Dose delivered	Registry	OHS
Dose received	Registry Questionnaire	OHS 305 POSE program participants
Fidelity	Interviews	2 OHS employees, 10 POSE program participants
Satisfaction	Questionnaire Interviews	305 POSE program participants 10 POSE program participants, 4 HR managers, 2 OHS employees
Context	Interviews	10 POSE program participants, 4 HR managers, 2 OHS employees

*POSE* promotion of sustained employability, *HR* human resource, *OHS* occupational health service

*Recruitment* refers to the strategies being used to recruit employees to participate in the POSE program. Information was retrieved from the OHS and HR management at the different plants. Reasons for non-participation were also evaluated during interviews.

*Reach* refers to the number of employees that participated in the POSE program. Reach was calculated by dividing the number of participants by the number of invited employees. Information was retrieved from OHS and company registry.

*Dose delivered* refers to the number of program components which were provided by the OHS to participants. Five program components were evaluated: questionnaire, biometry, FCE, counseling, and follow-up. These data were retrieved from OHS registry. Dose delivered was calculated for each component by dividing the number of participants a component was delivered to by the total number of participants.

*Dose received* refers to the number of program components which were actively received by program participants. Information was collected from OHS registry and by a questionnaire sent to all participants. Furthermore, during the interviews employees were also asked about the dose received.

*Fidelity* refers to the extent to which the program was delivered as planned and whether it was delivered according to protocol. Fidelity was evaluated by interviews with OHS representatives and POSE program participants.

*Satisfaction* refers to the extent to which participants were satisfied with separate program components. This was evaluated by questionnaire, 9 months after POSE program implementation. Participants were also asked whether they would recommend the program to colleagues. These questions could be answered on a numeric rating scale (NRS) ranging from 0 to 10. In addition to the questionnaires, three participants from each plant were interviewed to gain more in-depth insight in reasons for participating, satisfaction with the program (short-term and long-term), and recommendations for future implementation.

*Context* refers to factors in the organization, community, social/political context, or either situational issues that could potentially affect either intervention implementation or intervention outcome (barriers and facilitators). Context was evaluated by interviews with WHS participants, company and plant management, and OHS. Interviewees were asked to describe the entire process of preparation, implementation, and follow-up of the POSE program, and to highlight barriers and facilitators that could have affected implementation. Context was also addressed in the follow-up questionnaire which was distributed among participants, 9 months after the POSE program.

### Data Analysis

Quantitative data were analyzed using descriptive statistics. Furthermore, Fisher’s Exact test was performed to compare advice on follow-up between two age groups, under 50 years of age (n = 116) or 50 years and older (n = 189). The age distinction was based on recruitment strategies and on age distribution. In all analyses, SPSS for Windows version 22.0 was used (IBM Corp., Armonk, New York, USA). Interviews were audio recorded, with permission from interviewees, and transcribed verbatim. Subsequently, transcripts were read and reread by the first author and investigated on pre-specified themes. The analyses of the interviews with HR and OHS focused on contextual factors within and outside the organization that could have affected the implementation and execution of the POSE program. The interviews with participants focused on recruitment, dose received, satisfaction, and context.

## Results

### Sample Characteristics

Out of 986 eligible employees at the start of the study, 305 (31 %) participated in the POSE program. During the study two initially participating plants were closed due to economic reasons. A second group of employees from an already participating plant was enrolled in the study. Baseline characteristics of the participants are presented in Table 2. On average, employees were 50 years (SD 8.2) old when they participated in the POSE program, and had worked at the company for 22 years (SD 11.0). The majority of the participants was male (89 %). The follow-up questionnaire was filled out by 148 participants, and interviews were conducted with 4 HR managers, 2 OHS employees, and 10 POSE program participants.

**Table 2:** Baseline characteristics of POSE program participants

	Plant A	Plant B1	Plant B2	Plant C	Total
N	112	85	67	41	305
Age [year (mean, SD)]	47 (9.0)	48 (9.0)	55 (3.8)	53 (3.0)	50 (8.2)
Gender (% male)	91 %	93 %	93 %	68 %	89 %
Job tenure [year (mean, SD)]	22 (10.0)	20 (11.6)	24 (11.7)	20 (10.6)	22 (11.0)

### Process Evaluation

#### Recruitment, Reach

Different recruitment strategies were used between and within plants and different reach was attained (Table 3). At three plants all eligible employees were enrolled in the POSE program. They automatically participated unless they indicated otherwise. At the other plant employees could subscribe to the program voluntarily. At plants A and B1 the total contracted workforce was invited to participate in the POSE program. At plant B2 a smaller sample was invited, because this was the same plant as B1 and the sample was restricted to employees aged 50 years and older. Initially the whole workforce at plant C was eligible to participate, but this was later restricted to employees aged 50 years and older. Recruitment at plants B1 and B2 was done by the same person who indicated that the strategy to let employees automatically participate in the POSE program was preferred: “With this strategy employees did not have to take action, unless they did not want to participate. One way or the other, the barrier to decline participation is higher.” The employees from plant B1 who could not enter the POSE program due to restricted space could still enter the program in a later phase, because the program was repeated on a returning basis. The reach was calculated based on how many workers entered the POSE program, therefore 85 persons of a potential pool of 315 workers entered the program for this plant.

**Table 3:** Recruitment strategies and reach at the different plants

Location	Recruitment strategy	Reach^a^
Plant A	All contracted personnel were subscribed to the POSE program. Employees had to unsubscribe in case they did not want to participate	112/128 = 87.5 %
Plant B1	All contracted personnel were invited. Employees could subscribe themselves to the program. Place for approximately 80 participants	85/315 = 27.0 %
Plant B2	All contracted personnel of 50 years and older were subscribed to the POSE program, except employees that participated in the previous year. Employees had to unsubscribe in case they did not want to participate. Place for approximately 80 participants	67/90 = 74.4 %
Plant C	All contracted personnel of 50 years and older were subscribed to the POSE program, excluding participants from the previous year. Employees had to unsubscribe in case they did not want to participate	41/44 = 93.2 %
Total	–	305/577 = 52.9 %

^a^Reach = (participants/target sample) × 100 %

#### Dose Delivered, Dose Received, Fidelity

An overview of the dose delivered, dose received, and fidelity is presented in Table 4. The questionnaire and biometric measurements were actively delivered to and received by participants. They were all delivered according to protocol. Differences in delivery existed for FCE, because this was not delivered to the full extent at the different plants. FCE was purposefully not delivered to office personnel. Furthermore, FCE was offered to all production personnel, but not always received due to fulfillment of exclusion criteria (e.g. cardiovascular risk factors, musculoskeletal problems). This has caused differences in dose received between plants (66–94 %). In addition, FCE tests were not always administered according to protocol because tests were ended before one of the ending criteria was reached. Neither registration nor information from interviews with participants and OHS representatives were sufficient to assess fidelity of FCE. Counseling should have been delivered to every participant. However, although advices were mostly registered, it is unknown whether counseling was actually delivered to and received by participants. For these reasons dose delivered and dose received are regarded as not assessable. It is assumed that counseling did take place according to protocol in case it was delivered and received. Calling participants to check whether they had followed up on the advice they received during the POSE program was part of the planned follow-up. From interviews with OHS representatives it became clear that participants were called, but it was not registered how frequently this was done by the OHS. Therefore, dose delivered, dose received, and fidelity were evaluated as not assessable. Although the POSE program was not entirely performed according to protocol and there was little insight in interventions following the POSE program, the POSE program itself was perceived as the main intervention. To quote an OHS representative: “People already take action just by participating in the POSE program, so actually offering the POSE program is the main intervention.”

**Table 4:** Dose delivered, dose received, and fidelity of POSE program components

Component	Dose delivered^a^	Dose received^a^	Fidelity^a^
Questionnaire	100 (100–100)	95 (87–99)	100 (100–100)
Biometrics	100 (100–100)	96 (94–100)	100 (100–100)
FCE	90 (85–100)	81 (66–94)	n.a.
Counseling	n.a.	n.a.	100
Follow-up	n.a.	n.a.	n.a.

*n.a.* Not assessable

^a^Results are presented as mean percentage (range)

Although it is unknown whether all advices were delivered to and received by POSE program participants, the advices themselves were registered (Table 5). Five out of every six participants received advice on follow-up. They ranged from a visit to a general practitioner to workplace evaluations. Ten percent of the participants was already under treatment. Fisher’s Exact tests indicate that in general fewer advices were provided to workers aged under 50 (n = 116) compared to workers aged 50 and older (n = 189), although most differences were not significant. If differences were significant, the younger age category received advice less frequently. Furthermore, study questionnaires (n = 148) were analyzed on advices and follow-up. Ninety-nine participants indicated they had received advice during a counseling session of which 82 also had the intention to follow-up on the advice (score of 6 or higher on a 0–10 scale). The other forty-nine respondents either did not receive an advice or did not answer this question. Forty-nine respondents had already started or finished the follow-up, and 44 indicated not to have started (without providing a reason).

**Table 5:** Number of participants that received advice on follow-up after POSE program (N = 305), and comparison of age groups (<50 years, n = 116; > 50 years, n = 189)

Advice	n	%	% <50 years	% >50 years	p*
No advice	49	16	21	13	.11
Movement/exercise	87	29	26	30	.44
*General practitioner*	*83*	*27*	*15*	*35*	<*.01*
Dietician/nutrition	77	25	26	25	.78
Weight	72	24	22	24	.78
*Cholesterol*	*62*	*20*	*13*	*25*	<*.01*
*Smoking*	*56*	*18*	*12*	*22*	*.03*
Blood pressure	45	15	10	18	.10
*Visual*	*44*	*14*	*9*	*18*	*.03*
Audio	36	12	12	12	1.00
*Physical capacity*	*36*	*12*	*7*	*15*	*.04*
Physiotherapist, manual therapist, exercise therapist	33	11	8	13	.19
*Work ability*	*24*	*8*	*3*	*11*	<*.01*
Personal (mental) capacity	19	6	10	4	.09
Glucose	16	5	5	5	1.00
Occupational physician	14	5	2	6	.09
Workplace evaluation	13	4	3	5	.38
Alcohol use	7	2	2	3	.71
Relaxation	7	2	1	3	.26
Lung functioning	7	2	2	3	.71
Lifestyle	5	2	1	2	.65
Work stress	4	1	2	1	.64
Psychologist	1	0.3	0	0.5	1.00
Occupational social work	1	0.3	0.9	0	.38
*Already receiving treatment*	*29*	*10*	*3*	*13*	<*.01*

* Age comparison by Fisher’s Exact test between workers aged under 50 and workers aged 50 or older. Significant differences are in italics

#### Satisfaction

On a scale from 0 to 10 the participants appreciated the POSE program with a mean score of 7.6 (SD 1.1). The information that was provided to participants before the start of the POSE program was appreciated with a 7.4, ranging between plants from 6.9–7.8. The POSE program itself was awarded a 7.6 (range 7.3–7.8). The advice from the program was awarded an average of 7.7 (range 7.3–8.2). And the follow-up on the program received and average score of 7.7, ranging from 7.1 to 8.0. The majority of participants would recommend the POSE program to colleagues. Ninety-five percent of the questionnaire respondents (n = 135) indicated this by giving a grade of 6 or higher (scale 0–10).

POSE program participants were satisfied with the program and indicated that they felt it added value to their own health and employability. Main reasons for participation were strong interest in health, getting an update of one’s health status, but also because the employer stimulated participation. Two participants: “First I was a little skeptic, but when you start to think about it you realize that it might be valuable, mainly because you normally don’t consider things like this.” “Creating some awareness about lifestyle, that’s what they [the company] want to promote more or less.*”* Reasons for not participating were fear of bad outcomes, a feeling of the program being useless, or lack of interest. Two interviewees: “I think that some people knowingly did not participate because of a bad lifestyle, overweight, smoking, etc.” “Some colleagues said it was nonsense: if something is wrong with me I’ll go see a doctor.”

In the questionnaire 114 participants indicated what they found the most important outcome of the POSE program. Awareness of one’s health status was mentioned most frequently (n = 36; 32 %), followed by the measurement results (n = 34; 30 %), confirmation of one’s own perception (n = 12; 11 %), and advice (n = 12; 11 %).

#### Context

POSE program implementation started in February 2012, which was during economic recession in the Netherlands. The company closed two plants that were initially enrolled in the study. Those employees who were relocated to other plants were still eligible to participate in the POSE program.

An important motivator to implement the POSE program was the fact that Dutch law requires large employers to offer suitable occupational healthcare to employees. HR managers indicated that some difficulties were encountered during implementation at the first plants, which was partially caused by novelty of the POSE program, even though a pilot program already solved some teething troubles. Employees were still anxious about the consequences of the results. One manager suggested that more and better communication about the purpose of the program could have helped take away the anxiety. At that time, implementation of the POSE program was supported but enforced by company management and plant management was expected to execute the program. As one interviewee explained: “We actually forced it [the POSE program] to be deployed at the plants. It’s not the way we wanted to do it, but in this way we did give it a head start.”

Implementation of the POSE program resulted in some resistance among work floor supervisors who had to adjust planning. Not only because employees were away from work for some time, but also because the program took more time than they had anticipated. The long time between program execution and presentation of results at company level was considered a barrier for smooth follow-up of the POSE program. During that time participants were left unaware about follow-up at company level, for instance whether they were going to be invited to see an occupational physician, or were invited to participate in a company health program. And even afterwards it remained a vague process, because at the company no one was made responsible, costs were unknown, etcetera. One HR manager also mentioned that plant management did not receive feedback from the OHS regarding individual follow-up.

The POSE program was provided at a location near the workplace, during working time, which facilitated implementation and probably resulted in higher participation rates as well. Implementation became easier along the way. One interviewee indicated that over the past years societal influences created corporate responsibility and helped raise awareness among employees about responsibility for their own health. Both employer and employee became aware that they share responsibility regarding health and employability. Over time, the POSE program has been embedded in company policy and has even been expanded with other activities aimed at sustainable employability. Where personal employability was considered as something new and ‘scary’ to talk about, it is now a normal issue to discuss at the workplace and people want the employer to help them with their health problems. An employee confirmed this: “Well, at first people were a little distrusting towards the POSE program. Why is the company doing this, are they unsatisfied, do they want to change company culture? But then again I think it is a good sign towards employees that they want to listen to them.” And as another employee put it: “I believe it is a positive signal that the company makes employees aware and offers employees the opportunity to participate in the POSE program, and gives the opportunity to improve. Even though it is quite basic, you show corporate involvement.”

## Discussion

### Findings

This study is one of the first to investigate the implementation process of a workplace health promotion program. Our evaluation showed that the quality of program implementation varied. Different recruitment strategies were used which both seemed to be effective in attracting employees. POSE program questionnaire and biometrics were delivered and received as planned and were implemented according to protocol. However, FCE was not entirely delivered according to protocol, registration of counseling and follow-up was lacking and fidelity of specific components was not assessable. Nevertheless, participants were satisfied with the POSE program.

### Comparison to Other Studies

A number of other studies have reported on process evaluations of occupational healthcare. Among those studies two were conducted in the construction industry [9, 18], two in hospitals [10, 11], one in the financial sector [19], and one among workers with common mental disorders [20]. In our study different recruitment strategies (automatic enrollment, personal invitation) were used which resulted in different reach, i.e. from 27 to 93 %. Automatic enrollment to the POSE program resulted in higher participation rates. Other studies mainly used personal invitation strategies resulting in great variation in reach, ranging from 9 to 84 % [9, 10, 18, 19]. As mentioned in the results section, dose delivered and dose received could only be assessed for the questionnaire, biometrics and FCE. Fidelity was only assessable for the questionnaire, biometrics and counseling. Where assessable in our study, dose delivered ranged from 90 to 100 % which is similar to or higher than other studies in construction and mental healthcare, reporting delivered doses of 36–95 % [9, 18, 20]. Dose received was fairly high in our study, ranging from 81 to 96 %, where other studies reported lower values and more variation. In various target samples between 27 and 83 % dose received was reported [9, 10, 18, 20]. Fidelity in our study was estimated at 100 %, although not all intervention components could be evaluated due to missing or incomplete registration. Other studies reported lower (35 %) to equal values (100 %) [9, 10, 19]. Based on the high rates of dose delivered, dose received, and high fidelity one could assume that the POSE program has been implemented as it should have been. However, due to partial assessment of fidelity, this assumption should be treated with caution. Regarding participant satisfaction we found scores similar to other studies [9, 10, 18, 19]. Several contextual factors were identified in our study that either facilitated or hindered POSE program implementation. The economic situation formed a barrier in at least 2 plants, because they were shut down. Similar influences were reported for the construction industry [9, 18]. Management support facilitated implementation, which is in accordance with other studies [18, 21, 22]. Furthermore, Dutch collective labor agreements requiring employers to offer employees proper occupational healthcare also facilitated program implementation [9].

### Strength and Limitations

A particular strength of this study is the integration of quantitative and qualitative methods which provided us with information on the implementation process of the POSE program from different perspectives. Data were collected from organizational decision makers, participants in the study, and program suppliers. The quantitative approach provided insight into the amount and quality of program implementation and the qualitative approach allowed an in-depth view on contextual factors and satisfaction. Another strong aspect is the long time period between POSE program implementation and the interviews, which allowed follow-up and interventions to come to effect. On the other hand, this long period may have caused recall problems which may have led to the overlooking of some program elements during participant interview. This may have affected program results, but this did not affect program implementation. A limitation is the low response to the participant questionnaire, which might have been caused by low satisfaction with the POSE program or so called respondent fatigue. This may limit the validity of the results. A second limitation is that we developed our own process evaluation questionnaire. Although other validated questionnaires have been used to construct the questionnaire, several aspects had to be amended to suit the current research needs, and therefore may limit generalizability. Furthermore, purposive sampling, based on individual risk profiles, was applied to select POSE program participants for interviews. This strategy was followed to gain insight in different types of follow-up trajectories. It is unknown whether data saturation was reached, although the impression was that later interviews minimally added new information. It is possible that the applied sampling strategy did not result in a representative perspective of the WHS program, which may limit generalizability of the study results. A last limitation is that fidelity was only assessed during interviews. Although it did become clear that not everything was performed according to protocol, objective measures could have provided more detailed information on fidelity and hence the quality of program implementation.

## Conclusion and Recommendations

Knowledge on the performance on several process outcomes provides insight in reasons for success or failure of the implementation of a WHS program in the meat processing industry. The results of this process evaluation have shown that participants were satisfied with the POSE program and that the program raised awareness about health and employability. Furthermore, this study has shown that several contextual factors should be taken into account when implementing a WHS program. Organization at the workplace during paid working time facilitates participation, just as a positive attitude from company management. Even though the protocol was established before the study, not everything went according to plan. This study demonstrated that program implementation was sufficient regarding the online questionnaire and biometric screening. Delivery of FCE and delivery of the counseling session failed to some extent and could not always be evaluated due to absence of registration. These registration failures apply to follow-up measures as well. If the program is not effective on primary outcomes this can thus be attributed to program failure. Whether it might (also) be due to theory failure will be impossible to distinguish. Therefore, whether complete program compliance would lead to the intended effects cannot be foretold, but complete program compliance would be the first step. Some aspects can be improved, not only regarding implementation but also regarding evaluation. For instance, better administration and monitoring of program implementation might provide more insight in process aspects like fidelity. These findings show the relevance of performing a process evaluation in order to be able to adjust and improve program implementation in the future. New studies should take this into account.

## Appendix : Follow-Up Questionnaire

### Appendix: Interview with HR Managers and OHS Employees

1. How did you experience the workers’ health surveillance (WHS) program?
2. Can you describe the process of WHS implementation? Who were involved in this process, and how were things organized?
  - First of all, which processes occurred prior to the actual execution of the WHS program (organization, information, communication, etc.)
  - Subsequently, how was the WHS program deployed (execution and organization)?
  - Next, what was done in follow-up to the WHS program?
  - Can you think of factors that made it difficult to implement and execute the WHS program? Think of factors within the organization (company culture, (re)organization), but also outside the company (politics, economy, society).
    i. Could barriers be evaded, or solved?
    ii. How were these barriers solved?
  - Were there factors that catalyzed the implementation of the WHS program? If yes, which?
3. To which actions have the outcomes of the WHS program led? Were extra activities deployed to offer participants the opportunity to work on their ‘problem areas’?
  - If yes, what has been done?
  - How did people respond? How many people actually made use of these activities/facilities?
4. What are the following steps based on the WHS program?
  - How does the company secure that policies concerning ‘sustainable employability’ are embedded within the company?
5. What is your advice to the company, concerning ‘sustainable employability’?
  - What should the company do?
  - What could or should you do?

### Appendix: Interview with POSE Program Participants

1. Some time ago your company has implemented a workers’ health surveillance (WHS) program. Regarding the WHS program, how have you experienced that?
  - Can you mention some good aspects?
  - Which aspects were less good, or maybe even negative?
  - Why did you participate in the WHS program?
    i. Do you know why colleagues participated, or why not?
  - Do you think the company put in a lot of effort to inform people about the program and to offer the opportunity to participate?
    i. How much effort did the company put into keep people involved?
    ii. How did the management team respond?
2. What happened after the WHS program?
  - Do you think it has caused changes in the company? And how would you describe these changes? (for instance, company culture/health activities/etc.)
  - What did the company do in the past period? Does this meet your expectations?
  - What has been achieved with the WHS program?
  - In your opinion, what is the greatest benefit of the program?
  - If the program were to continue, what would you change?
3. Is there anything else about the WHS program you would like to share?
